# Pathological outcomes in women with cervical adenocarcinoma *In Situ* treated by conisation or conisation followed by hysterectomy

**DOI:** 10.3389/fonc.2026.1692524

**Published:** 2026-01-27

**Authors:** Lan Ying Li, Da Long Song, Xiao Ying Sun, Zhi Gang Li, Ke Li You

**Affiliations:** Department of Gynecology, Guangdong Provincial People’s Hospital, Guangdong Academy of Medical Sciences, Southern Medical University, Guangzhou, Guangdong, China

**Keywords:** adenocarcinoma in situ, hysterectomy, pathological outcome, residual lesion, treatment

## Abstract

**Objective:**

This study aimed to evaluate the pathological outcomes of conisation with or without subsequent hysterectomy in patients diagnosed with adenocarcinoma *in situ* (AIS) and grade 3 cervical intraepithelial neoplasia (CIN3), emphasizing the impact of margin status on residual disease and recurrence risk.

**Methods:**

A retrospective analysis was conducted on patients diagnosed with AIS and CIN3 who underwent loop electrosurgical excision procedure (LEEP) or cold-knife conisation (CKC) between January 2012 and December 2022. The pathological findings of conisation and subsequent hysterectomy, as well as recurrence rates in patients managed conservatively, were analyzed. Patients were followed for a minimum of 3 years, with recurrence defined as the detection of cervical intraepithelial neoplasia (CIN) through colposcopy.

**Results:**

A total of 387 patients were included: 107 with AIS and 280 with CIN3. Hysterectomy was performed in 72.9% of AIS patients and 49.3% of CIN3 patients after conisation. Positive conisation margins were associated with a higher likelihood of residual disease in hysterectomy specimens, especially in cases with cervical canal involvement in AIS (70.0%) and with combined endo/ectocervical and canal margin involvement in CIN3 (60.0%). In multivariable logistic regression analysis adjusting for age, HPV status, and conisation technique, cervical canal margin involvement was independently associated with positive hysterectomy pathology in AIS (OR 6.962, 95% CI 1.393–34.794; p = 0.018), whereas endo/ectocervical margin involvement was the independent predictor in CIN3 (OR 6.790, 95% CI 1.683–27.387; p = 0.007). The preoperative HPV infection rate was lower in AIS than in CIN3 (88.8% vs 97.1%, p = 0.002).

**Conclusion:**

While hysterectomy is recommended for AIS with positive margins, conisation alone may be a safe alternative for selected patients without involvement of the cervical canal margin.

## Introduction

In recent decades, while the incidence of squamous cell carcinomas has diminished, the global prevalence of adenocarcinoma has seen a notable rise (1–3). Adenocarcinoma *in situ* (AIS) of the uterine cervix is regarded as a precursor lesion with the potential to progress to invasive cervical adenocarcinoma (4). Among women undergoing cervical cancer screening, the incidence of AIS has escalated significantly, particularly in those within the 30–39 age group (2, 5, 6).

Traditionally, most European and American guidelines advocate simple hysterectomy as the preferred treatment for AIS, as it ensures complete removal of potentially precancerous tissue (7–9). Despite relatively low reported recurrence rates, these guidelines and studies (10) advise a subsequent hysterectomy following conservative management once childbearing is no longer a concern. Similarly, Japanese guidelines recommend simple hysterectomy for AIS with negative margins. Fertility preservation is considered only in cases where the patient expresses a strong desire to maintain reproductive potential, necessitating meticulous evaluation and close monitoring (11).

In recent years, the oncological safety of conservative management for AIS using conisation has been reassessed. A recent study by Munro et al. reported a 2.8% risk of recurrent AIS after conservative treatment with initially negative histological margins over a mean follow-up of 3.9 years (12). While phase III clinical trials are not available, a substantial body of evidence suggests that this approach may serve as a viable and oncological safe option (13–19). In this context, analyzing the pathological outcomes of hysterectomy following conisation holds significant clinical value in evaluating the efficacy of conisation as a standalone treatment for AIS, even in patients without fertility preservation needs.

In this context, evaluating pathological outcomes in hysterectomy specimens following conisation provides important insight into the adequacy of conservative treatment. Furthermore, including CIN3 as a comparative high-grade squamous precursor lesion—commonly managed with conisation—allows assessment of whether margin status confers a differential risk of residual disease between glandular and squamous lesions, thereby adding clinically relevant context beyond existing single-entity studies.

## Materials and methods

### Study population and data collection

This study retrospective study was approved by the Institutional Review Board of Guangdong Provincial People’s Hospital (Approval Number: KY2024-981-01). All patient data were anonymized to ensure confidentiality.

The study included women diagnosed with cervical adenocarcinoma *in situ* (AIS) and grade 3 cervical intraepithelial neoplasia (CIN3), who underwent treatment between January 2012 and December 2022. The inclusion criteria were as follows: 1) Histopathological confirmation of AIS, CIN3.2) All cases should undergo either loop electrosurgical excision procedure (LEEP) or cold-knife conisation (CKC) for diagnosis and treatment.3) Sufficient clinical and pathological data available for analysis. Exclusion Criteria were as follows: 1) Cases diagnosed with invasive adenocarcinoma following LEEP or CKC.) Patients with incomplete medical records or loss to follow-up.

All patients underwent either LEEP or CKC as the initial excisional procedure. The choice between conservative management and hysterectomy was guided by the treating physician, taking into account clinical indications, lesion characteristics, patient age, fertility preservation preferences, and comorbidities. LEEP was executed using a wire loop electrode under colposcopic visualization to precisely excise the transformation zone. CKC, on the other hand, involved the removal of a conical cervical specimen using a cold scalpel, generally performed under general anesthesia to ensure patient comfort and procedural accuracy. The focus was on the status of the resection margins after conisation, including whether the ectocervical margins and endocervical margins were positive. Patients with positive margins after the first conisation who achieved negative margins through a subsequent repeat conization were also considered as having negative margins. At the same time, the pathological results of the subsequent hysterectomy specimens were also carefully examined to determine if there were residual lesions. All the hysterectomies were performed within 6 months after the conisation procedure. Recurrence was defined as the detection of cervical intraepithelial neoplasia (CIN) by colposcopy. Patients were followed up regularly, usually including physical examinations, cervical cytology tests, and colposcopy examinations at specific time intervals. The follow - up duration varied among patients, but the minimum follow - up period was 3 years to ensure the detection of potential recurrence.

Histopathological evaluation was performed by experienced gynecologic pathologists according to institutional standards. For diagnostically challenging cases, slides were reviewed by at least two pathologists to reach a consensus.

In this study, recurrence was defined solely as the detection of CIN on colposcopy during follow-up; HPV persistence was not incorporated into the recurrence criteria after treatment.

### Statistical analysis

All statistical analyses were performed using SPSS version 29.0.1.0 (IBM Corp., Armonk, NY). Descriptive statistics were used to summarize baseline characteristics. Comparisons between groups were conducted using chi-square or Fisher’s exact tests for categorical variables and t-tests or Mann-Whitney U tests for continuous variables. Variables with clinical relevance were subsequently included in a multivariable logistic regression model. The model was adjusted for age, HPV status, conisation technique (LEEP vs CKC), and margin status. Separate multivariable analyses were conducted for AIS and CIN3. (ORs) with 95% confidence intervals (CIs) were reported. A two-sided *P* value < 0.05 was considered statistically significant. No adjustment for multiple comparisons was performed, given the exploratory nature of the study. All the statistical analyses were conducted using SPSS Statistics version 29.0.1.0 (IBM, Armonk, NY.USA).

## Results

### Patient characteristics

This study enrolled patients diagnosed with cervical adenocarcinoma *in situ* (AIS) (N = 107) and grade 3 cervical intraepithelial neoplasia (N = 280) at our institution between January 2012 and December 2022.The median age of patients at the time of the first conisation was 43 years (range: 24–73) in the AIS group and 46 years (range: 22–72) in grade 3 cervical intraepithelial neoplasia group. LEEP was more frequently performed in CIN3 patients (85.7%) than in AIS patients (70.1%), whereas AIS patients were more likely to undergo CKC (29.9% vs. 14.3%, *P* < 0.001). Hysterectomy was performed more frequently in the AIS group than in the CIN3 group (72.9% vs. 49.3%, *P* < 0.001). In terms of HPV status, the preoperative HPV infection rates were 88.8% and 97.1% in the AIS and CIN3 group. The HPV infection rate in the AIS group was significantly lower than that CIN3 group (P = 0.002) (**Table 1**).

**Table 1:** Characteristics of AIS and CIN3 patients(N=387).

Characteristics	AIS	CIN3	P Value
	(N =107)	(N =280)	
Age, median (range), y	43(24-73)	45.7(22-72)	0.026
Operation mode, No. (%)			
LEEP	75(70.1%)	240(85.7%)	<0.001
CKC	32(29.9%)	40(14.3%)	
Hysterectomy case (%)			
No	29(27.1%)	142(50.7%)	<0.001
Yes	78(72.9%)	138(49.3%)	
HPV infection			
No	12(11.2%)	8(2.9%)	0.002
Yes	85(88.8%)	272(97.1%)	

AIS, adenocarcinoma in situ; CIN3, grade 3 cervical intraepithelial neoplasia; LEEP, loop electrosurgical excision procedure; CKC, cold-knife conisation.

### Pathological results of conisation and hysterectomy

Among patients who underwent conisation followed by hysterectomy, the incidence of positive findings in hysterectomy pathology varied by conisation margin status and lesion type (**Table 2**). For patients diagnosed with AIS, those with positive conisation margins showed different rates of positive hysterectomy pathology depending on the affected area: 15.4% (2/13) for endo/ectocervical involvement, 70.0% (7/10) for cervical canal involvement, and 57.9% (11/19) for both. In contrast, patients with negative margins had a lower rate of 13.9% (5/36). In multivariable logistic regression analysis, adjusting for age, HPV status, and conisation technique (LEEP vs CKC), cervical canal margin involvement was independently associated with positive hysterectomy pathology in AIS patients (OR 6.96, 95% CI 1.393–34.794, p = 0.018), whereas endo/ectocervical margin involvement was not (**Table 3**, **Supplementary Table 1**). None of the AIS patients experienced recurrence during follow-up.

**Table 2:** Incidence of positive findings in hysterectomy pathology and follow-up period.

	Groups	Conisation Margin		N	Positive Hysterectomy Pathology % (N)	*Recurrence Number	Median follow-up period (Months, IQR)	DFS(Months)
Conisation+Hysterectomy	AIS	+	Endo/Ectocervical	13	15.4%(2/13)	0	26 months (15-44)	
			Cervical canal	10	70.0%(7/10)	0	35.5 months (14.5-49.5)	
			Both	19	57.9%(11/19)	0	30 months (10.5-58.5)	
		–		36	13.9%(5/36)	0	38.5 months (19-76.5)	
	CIN3	+	Endo/Ectocervical	34	50.0%(17/34)	0	21 months (13.8-60)	
			Cervical canal	20	35.0%(7/20)	0	21.5 months (13.5-46)	
			Both	55	60.0%(33/55)	0	40.5 months (21-60.5)	
		–		29	20.7%(6/29)	1	42 months (18-62)	147

*Recurrence was defined as the detection of CIN by colposcopy.

**Table 3:** Multivariable logistic regression analysis of factors associated with positive hysterectomy pathology.

Pathology Type	Margin status	OR(95%CI)	P Value
AIS	Endo/Ectocervical margin		
	Negative	1 (reference)	0.761
	Positive	0.732(0.098-5.443)	
	Canal margin		
	Negative	1 (reference)	0.018
	Positive	6.962(1.393-34.794)	
CIN3	Endo/Ectocervical margin		
	Negative	1 (reference)	0.007
	Positive	6.790(1.683-27.387)	
	Canal margin		
	Negative	1 (reference)	0.13
	Positive	3.241(0.706-14.873)	

For patients with CIN3, positive hysterectomy pathology was observed in 50.0% (17/34) of those with endo/ectocervical involvement, 35.0% (7/20) of those with cervical canal involvement, and 60.0% (33/55) of those with both. Negative margin cases had a much lower incidence of positive findings (20.7%, 6/29) with one case recurrence. In the multivariable logistic regression model, adjusting for age, HPV status, and conisation technique (LEEP vs CKC), CIN3 patients with endo/ectocervical margin involvement showed a significantly increased likelihood of positive hysterectomy pathology (OR 6.79, 95% CI 1.683–27.387; *p* = 0.007), while cervical canal margin involvement did not reach statistical significance (**Table 3**, **Supplementary Table 2**).

In patients undergoing conisation alone, follow-up results varied by lesion type and margin status. For AIS patients, none of the patients with either positive or negative margins had recurrence during follow-up (**Supplementary Table 3**). In this group, the median follow-up duration for patients with negative margins was 51 months (IQR, 15.5–77.0). Among patients with endo-/ectocervical margin involvement (n = 2), the follow-up durations were 162 and 119 months, respectively. For those with cervical canal margin involvement (n=2), the follow-up durations were 35 and 64 months. Patients with involvement of both margin sites(n=3) had follow-up of 28, 92, and 159months, respectively. For CIN3 patients, the median follow-up period was 34.0 months (IQR, 15.3–53.5) for those with negative margins. Among patients with positive margins, the median follow-up was 26.0 months (IQR, 12.0–53.0) for endo-/ectocervical involvement, 41.0 months (IQR, 29.0–92.0) for cervical canal involvement, and 42.5 months (IQR, 26.5–56.5) for involvement of both areas. Three recurrences were observed among CIN3 patients with endocervical or/and ectocervical positive margins, with DFS of 22, 12, and 4months, respectively. One recurrence was observed in a patient with both endo-ectocervical and cervical canal positive margins, with a DFS of 48 months. One recurrence was observed in a patient with negative margins, with a DFS of 52 months (**Supplementary Table 3**). These findings highlighted the impact of margin status on the likelihood of residual disease and recurrence risk, emphasizing the importance of complete excision in patients undergoing conisation.

## Discussion

This study provides valuable insights into the outcomes of conisation and subsequent hysterectomy in patients diagnosed with AIS through a comparative analysis of AIS with CIN3. Our findings reinforce the importance of conisation margin status in predicting residual disease and highlight the potential role of conisation as a standalone treatment in AIS patients.

In our study, patients with AIS were diagnosed at a younger age and underwent hysterectomy more frequently than those with CIN3, reflecting the clinical concern regarding the higher risk of residual or recurrent disease in AIS. The greater use of CKC in AIS also highlighted the importance of achieving clear margins in glandular lesions, which are often located higher in the endocervical canal and are more difficult to evaluate than squamous lesion. Unlike CIN3, which is almost universally driven by HPV infection, the development of AIS appears to involve some additional or alternative factors. For younger patients, treatment must therefore be individualized to effectively eliminate risk while conserving reproductive potential.

Our results demonstrate that positive conisation margins are associated with a higher incidence of residual disease in hysterectomy specimens both in AIS and CIN3 groups. However, the incidence varied according to the specific margin involved. Among AIS patients, those with cervical canal involvement had the highest rate of positive pathology in hysterectomy specimens (70.0%), whereas patients with positive endo/ectocervical margins (15.4%) and those with negative margins (13.9%) showed similarly much lower rates. This finding was further supported by multivariable logistic regression analysis, in which cervical canal margin involvement remained independently associated with positive hysterectomy pathology after adjustment for age, HPV status, and conisation technique (LEEP vs CKC), highlighting cervical canal involvement as a particularly high-risk margin status in AIS. These findings suggest that positive margins, particularly in the cervical canal, may indicate a higher likelihood of residual disease in AIS, reinforcing the need for further treatment such as second-conisation or hysterectomy in these cases. In contrast, lesions limited to the endo/ectocervical margins appear to be more effectively managed with conization, resulting in a substantially lower risk of residual disease in hysterectomy specimens. In addition, only two AIS patients with positive margins underwent a second conisation, which achieved negative margins and were not associated with recurrence. Similar findings were reported in the Nationwide cohort study, which demonstrated that when a radical excision is achieved, conservative treatment for AIS can be considered a safe and definitive treatment option (20).

In the CIN3 group, positive margins were strongly associated with residual disease in hysterectomy specimens, reaching 60.0% when both the endo/ectocervical and canal margins were involved, and 50.0% even when limited to the endo/ectocervical margins. In contrast, patients with negative margins showed a much lower residual disease rate of 13.9%. This association was further confirmed in multivariable logistic regression analysis, in which endo/ectocervical margin involvement remained an independent predictor of positive hysterectomy pathology after adjustment for age, HPV status, and conisation technique. These findings underscore the importance of margin status as a critical determinant in guiding the need for further intervention.

A key question in the management of AIS is whether conisation alone can be a safe and effective treatment option. Our results indicate that selected AIS patients, particularly those without cervical canal involvement, may be safely managed with conisation alone, consistent with recent meta-analyses (21)demonstrating favorable oncological outcomes after conservative treatment. However, our findings should be interpreted with appropriate caution owing to the limited sample size and the retrospective nature of the study. The disease-free survival (DFS) in AIS patients was particularly promising, with negative margin cases reaching 51.0. CIN3 patients with negative margin had DFS of 52 months, with one recurrence observed. These results align with recent studies (15, 20–23) suggesting that conisation alone may be sufficient for carefully selected AIS patients, particularly those with negative margins. Although the number of AIS cases with positive margins treated by conization alone was small in our cohort, hysterectomy pathology indicated that even conizations with endo/ectocervical positive margins might be safely managed without immediate further surgery.

In contrast, in the CIN3 group, four patients with positive margins experienced recurrence during follow-up, and one recurrence was observed in a patient with negative margins. This suggests that while conisation may be a viable option for some CIN3 patients, it carries a higher risk of recurrence particularly when margins are positive. Therefore, individualized treatment strategies should be considered, balancing the risks of recurrence with the patient’s preferences and overall health condition.

Although the HPV infection rate was lower in AIS than in CIN3 in our cohort, the majority of AIS cases were still HPV-positive. This finding is consistent with existing evidence that high-risk HPV infection plays a central role in the pathogenesis of AIS (3, 24–27). The observed difference in HPV prevalence between AIS and CIN3 may be partly explained by differences in sample size, patient selection, HPV testing methods over the long study period, and the inclusion of HPV-independent glandular lesions, which have been increasingly recognized in recent years. In addition, missing HPV genotype information in a subset of patients may have contributed to an underestimation of HPV positivity in the AIS group. Therefore, this finding should be interpreted with caution.

Our findings highlight the need for a tailored approach in the management of AIS and CIN3. For patients with positive margins, especially in the cervical canal, hysterectomy should be strongly considered due to the higher likelihood of residual disease. However, for patients with negative margins, particularly in the AIS group, conisation alone may be a reasonable option, provided that regular follow-up is maintained. These findings may have implications for current clinical guidelines, such as those from the SGO, ESGO, and JSGO, by supporting a more individualized approach to AIS management based on conisation margin status and fertility preservation considerations. Our summary **Table 4** is intended to reflect the findings of the present study and does not replace existing clinical guidelines. Clinical decisions should be individualized based on patient characteristics and preferences.

**Table 4:** Proposed clinical decision summary based on conisation margin status.

Pathological type	Conization Margin		Suggested strategy
AIS	Positive	Endo/Ectocervical	Repeat conisation
		Cervical canal	hysterectomy
	Negative		conisation alone & close follow-up
CIN3	Positive	Endo/Ectocervical	Repeat conisation/hysterectomy
		Cervical canal	Repeat conisation/hysterectomy
	Negative		conisation alone & close follow-up

This study has several limitations. Its retrospective, single-center design may have introduced selection bias, as treatment decisions were influenced by clinical judgment, patient characteristics, and fertility considerations. The long inclusion period (2012–2022) may have resulted in temporal heterogeneity due to changes in surgical techniques, pathological assessment, HPV testing, and follow-up strategies. In addition, the number of AIS cases was limited, particularly among patients with positive margins managed conservatively, which restricted statistical power and precluded detailed subgroup analyses. Recurrence was defined by histological confirmation at follow-up colposcopy; however, most recurrent lesions were squamous in nature, and no specific assessment of glandular persistence was performed. Moreover, the follow-up duration may not have been sufficient to capture late recurrences of glandular disease. Therefore, the results should be interpreted with caution, and larger prospective multicenter studies with standardized protocols and longer follow-up are warranted.

## Conclusion

This study underscores the critical role of different margin status in determining the risk of residual disease and recurrence following conisation. While hysterectomy remains the standard treatment for AIS, our findings suggest that conisation alone may be a safe option in carefully selected cases, particularly those without cervical canal involvement. Individualized patient management, guided by margin status and close follow-up, is essential to optimizing treatment outcomes while minimizing unnecessary surgical interventions.

## Data Availability

The raw data supporting the conclusions of this article will be made available by the authors, without undue reservation.

## References

[1] Adegoke O Kulasingam S Virnig B . Cervical cancer trends in the United States: a 35-year population-based analysis. J Womens Health (Larchmt). (2012) 21:1031–7. doi: 10.1089/jwh.2011.3385, PMID: 22816437 PMC3521146

[2] van der Horst J Siebers AG Bulten J Massuger LF de Kok IM . Increasing incidence of invasive and in *situ* cervical adenocarcinoma in the Netherlands during 2004-2013. Cancer Med. (2017) 6:416–23. doi: 10.1002/cam4.971, PMID: 28102052 PMC5313636

[3] Park KJ . Cervical adenocarcinoma: integration of HPV status, pattern of invasion, morphology and molecular markers into classification. Histopathology. (2020) 76:112–27. doi: 10.1111/his.13995, PMID: 31846527

[4] Zaino RJ . Symposium part I: adenocarcinoma in *situ*, glandular dysplasia, and early invasive adenocarcinoma of the uterine cervix. Int J Gynecol Pathol. (2002) 21:314–26. doi: 10.1097/00004347-200210000-00002, PMID: 12352181

[5] Cleveland AA Gargano JW Park IU Griffin MR Niccolai LM Powell M . Cervical adenocarcinoma in *situ*: Human papillomavirus types and incidence trends in five states, 2008-2015. Int J Cancer. (2020) 146:810–8. doi: 10.1002/ijc.32340, PMID: 30980692 PMC9112013

[6] Monti E Somigliana E Alberico D Boero V Iorio M Di Loreto E . Conservative treatment for cervical adenocarcinoma *in situ*: long-term results. J Low Genit Tract Dis. (2022) 26:293–7. doi: 10.1097/LGT.0000000000000688, PMID: 35917498

[7] Teoh D Musa F Salani R Huh W Jimenez E . Diagnosis and management of adenocarcinoma in situ: A society of gynecologic oncology evidence-based review and recommendations. Obstet Gynecol. (2020) 135:869–78. doi: 10.1097/AOG.0000000000003761, PMID: 32168211 PMC7098444

[8] McGee AE Alibegashvili T Elfgren K Frey B Grigore M Heinonen A . European consensus statement on expert colposcopy. Eur J Obstet Gynecol Reprod Biol. (2023) 290:27–37. doi: 10.1016/j.ejogrb.2023.08.369, PMID: 37716200

[9] Perkins RB Guido RS Castle PE Chelmow D Einstein MH Garcia F . 2019 ASCCP risk-based management consensus guidelines for abnormal cervical cancer screening tests and cancer precursors. J Low Genit Tract Dis. (2020) 24:102–31. doi: 10.1097/LGT.0000000000000525, PMID: 32243307 PMC7147428

[10] Le Gac M Benoit L Koual M Bentivegna E Bolze PA Kerbage Y . Exploring uterine involvement in hysterectomy samples following conization for adenocarcinoma in *situ* of the uterine cervix: Insights from a multicenter study by the FRANCOGYN group. J Gynecol Obstet Hum Reprod. (2024) 53:102826. doi: 10.1016/j.jogoh.2024.102826, PMID: 39074662

[11] Seino M Nagase S Tokunaga H Yamagami W Kobayashi Y Tabata T . Japan Society of Gynecologic Oncology 2022 guidelines for uterine cervical neoplasm treatment. J Gynecol Oncol. (2024) 35:e15. doi: 10.3802/jgo.2024.35.e15, PMID: 38037547 PMC10792216

[12] Munro Aime Codde Jim Spilsbury Katrina Stewart Colin JR Steel Nerida Leung Yee . Risk of persistent or recurrent neoplasia in conservatively treated women with cervical adenocarcinoma in *situ* with negative histological margins. Acta Obstet Gynecol Scand. (2017) 96:432–7. doi: 10.1111/aogs.13110, PMID: 28181670

[13] Salani R Puri I Bristow RE . Adenocarcinoma in *situ* of the uterine cervix: a metaanalysis of 1278 patients evaluating the predictive value of conization margin status. Am J Obstet Gynecol. (2009) 200:182.e181–185. doi: 10.1016/j.ajog.2008.09.012, PMID: 19019325

[14] Kim Mi-La Hahn Ho-Suap Lim Kyung-Taek Lee Ki-Heon Kim Hy-Sook Hong Sung-Ran . The safety of conization in the management of adenocarcinoma in *situ* of the uterine cervix. J Gynecol Oncol. (2011) 22:25–31. doi: 10.3802/jgo.2011.22.1.25, PMID: 21607092 PMC3097330

[15] Adolph L Mann A Liu X Q Roberts L Robinson C Popowich S . Follow-up of women with cervical adenocarcinoma in *situ* treated by conization: A single centre clinical experience. Gynecol Oncol. (2024) 187:74–9. doi: 10.1016/j.ygyno.2024.05.004, PMID: 38733955

[16] Schaafsma M Schuurman TN Siebers AG Bekkers RLM Bleeker MCG Zusterzeel PLM . Cost-effectiveness of an additional hysterectomy after initially conservative treatment for cervical adenocarcinoma. situ. Gynecol Oncol. (2025) 193:113–8. doi: 10.1016/j.ygyno.2025.01.005, PMID: 39826428

[17] Liu Jingjing Wang Yu Wan Xiaoyun Zou Jian Zhu Yedan Lv Weiguo . Comparison of the safety between cervical conization and hysterectomy for patients with cervical adenocarcinoma. situ. J Gynecol Oncol. (2023) 34:e8. doi: 10.3802/jgo.2023.34.e8, PMID: 36424703 PMC9807369

[18] Soegaard-Andersen E Frandsen AP Sandahl P . Adenocarcinoma *in situ* of the uterine cervix (AIS) treated by loop electrosurgical excision procedure strategy: an observational study. J Low Genit Tract Dis. (2024) 28:149–52. doi: 10.1097/LGT.0000000000000797, PMID: 38251975

[19] Zeng R Guo Y Zou M Wang C Wu X . CIN coexisting with AIS is a risk factor for residual disease after conization for cervical adenocarcinoma. situ. Front Oncol. (2025) 15:1571130. doi: 10.3389/fonc.2025.1571130, PMID: 40552263 PMC12183795

[20] Schaafsma M Schuurman TN Kootstra P Issa D Hermans I Bleeker MCG . Nationwide cohort study on the risk of high-grade cervical dysplasia and carcinoma after conservative treatment or hysterectomy for adenocarcinoma. situ. Int J Cancer. (2025) 156:1203–12. doi: 10.1002/ijc.35237, PMID: 39495176 PMC11736995

[21] Delli Carpini G Cicoli C Bernardi M Di Giuseppe J Giannella L Ciavattini A . Clinical outcomes of cervical adenocarcinoma in situ according to conservative or demolitive treatment: A systematic review and meta-analysis. Cancers (Basel). (2025) 17:1839. doi: 10.3390/cancers17111839, PMID: 40507318 PMC12153624

[22] Tan Jeffrey H J Malloy Michael J Thangamani Ramaish Gertig Dorota Drennan Kelly T Wrede C David . Management and long-term outcomes of women with adenocarcinoma in *situ* of the cervix: A retrospective study. Aust N Z J Obstet Gynaecol. (2020) 60:123–9. doi: 10.1111/ajo.13047, PMID: 31578727

[23] Ding HJ Zhang YY Li M Guo HY Zhang K He HJ . Risk factors predicting positive surgical margins following conization and residual disease in subsequent hysterectomy among postmenopausal women with cervical intraepithelial neoplasia. Ann Ital Chir. (2025) 96:905–15. doi: 10.62713/aic.4075, PMID: 40665947

[24] Schiffman Mark Mirabello Lisa Egemen Didem Befano Brian Xiao Yanzi Wentzensen Nicolas . The combined finding of HPV 16, 18, or 45 and cytologic Atypical Glandular Cells (AGC) indicates a greatly elevated risk of in *situ* and invasive cervical adenocarcinoma. Gynecol Oncol. (2023) 174:253–61. doi: 10.1016/j.ygyno.2023.05.011, PMID: 37243996 PMC11089431

[25] Costa Silvano Venturoli Simona Origoni Massimo Preti Mario Mariani Luciano Cristoforoni Paolo . Performance of HPV DNA testing in the follow-up after treatment of high-grade cervical lesions, adenocarcinoma in *situ* (AIS) and microinvasive carcinoma. Ecancermedicalscience. (2015) 9:528. doi: 10.3332/ecancer.2015.528, PMID: 25987897 PMC4431402

[26] Giannella L Delli Carpini G Di Giuseppe J Bogani G Sopracordevole F Clemente N . In situ/microinvasive adenocarcinoma of the uterine cervix and HPV-type impact: pathologic features, treatment options, and follow-up outcomes-cervical adenocarcinoma study group (CAS-group). Cancers (Basel). (2023) 15:2876. doi: 10.3390/cancers15112876, PMID: 37296839 PMC10252050

[27] Giannella L Di Giuseppe J Delli Carpini G Grelloni C Fichera M Sartini G . HPV-negative adenocarcinomas of the uterine cervix: from molecular characterization to clinical implications. Int J Mol Sci. (2022) 23:15022. doi: 10.3390/ijms232315022, PMID: 36499345 PMC9735497

