# Supplementary material for: Pathological outcomes in women with cervical adenocarcinoma In Situ treated by conisation or conisation followed by hysterectomy
Source: Front Oncol. 2026 Jan 27;16:1692524. doi: 10.3389/fonc.2026.1692524 (PMC12886004; doi:10.3389/fonc.2026.1692524)
Supplement: Supplementary file 1 [file Table1.docx]

Supplementary table 1. Multivariable logistic regression analysis of cervical canal margin involvement and positive hysterectomy pathology in AIS

| Variable | OR（95%CI） | P Value |
| --- | --- | --- |
| Age | 1.059（0.977-1.148） | 0.163 |
| Conisation technique |  |  |
| LEEP | 1 (reference) | 0.884 |
| CKC | 0.875（0.148-5.194） |  |
| **Canal margin** |  |  |
| Negative | 1 (reference) | 0.018 |
| Positive | 6.962（1.392-34.794） |  |
| HPV infection |  |  |
| No | 1 (reference) | 0.518 |
| Yes | 0.518（0.057-4.686） |  |
