# Supplementary material for: Pathological outcomes in women with cervical adenocarcinoma In Situ treated by conisation or conisation followed by hysterectomy
Source: Front Oncol. 2026 Jan 27;16:1692524. doi: 10.3389/fonc.2026.1692524 (PMC12886004; doi:10.3389/fonc.2026.1692524)
Supplement: Supplementary file 2 [file Table2.docx]

Supplementary table 2. Multivariable logistic regression analysis of endo/ectocervical margin involvement and positive hysterectomy pathology in CIN3

| Variable | OR（95%CI） | P Value |
| --- | --- | --- |
| Age | 1.005(0.946-1.067) | 0.871 |
| Conisation technique |  |  |
| LEEP | 1 (reference) | 0.036 |
| CKC | 6.741(1.129-40.254) |  |
| **Endo/ectocervical margin** |  |  |
| Negative | 1 (reference) | 0.007 |
| Positive | 6.790(1.683-27.387) |  |
| HPV infection |  |  |
| No | 1 (reference) | 0.691 |
| Yes | 0.568(0.035-9.217) |  |
