# Supplementary material for: Pathological outcomes in women with cervical adenocarcinoma In Situ treated by conisation or conisation followed by hysterectomy
Source: Front Oncol. 2026 Jan 27;16:1692524. doi: 10.3389/fonc.2026.1692524 (PMC12886004; doi:10.3389/fonc.2026.1692524)
Supplement: Supplementary file 3 [file Table3.docx]

Supplementary Table3. Recurrence during follow-up in the conization alone group

| **Conisation Pathology** | **Conisation Margin** | | **N** | ***Recurrence Number** | **Median follow-up period (Months,IQR)** | **DFS（Months ）** |
| --- | --- | --- | --- | --- | --- | --- |
| AIS | + | Endo/Ectocervical | 2 | 0 | 162,119 |  |
|  |  | Cervical canal | 2 | 0 | 35,64 |  |
|  |  | Both | 3 | 0 | 28,92,159 |  |
|  | - | | 22 | 0 | 51(15.5-77) |  |
| CIN3 | + | Endo/Ectocervical | 25 | 3 | 26(12-53) | 22, 12,4 |
|  |  | Cervical canal | 13 | 0 | 41(29-92) |  |
|  |  | Both | 30 | 1 | 42.5(26.5-56.5) | 48 |
|  | - | | 74 | 1 | 34(15.3-53.5) | 52 |
